# Analysis of local protein accumulation kinetics by live-cell imaging in yeast systems

**DOI:** 10.1016/j.xpro.2021.100733

**Published:** 2021-08-17

**Authors:** Hiroki Okada, Brittany MacTaggart, Erfei Bi

**Affiliations:** 1 Department of Cell and Developmental Biology, Perelman School of Medicine, University of Pennsylvania, Philadelphia, PA 19104-6058, USA

**Keywords:** Cell Biology, Genetics, Microscopy, Model Organisms

## Abstract

Microscopy-based analysis of protein accumulation at a given subcellular location in real time provides invaluable insights into the function of a protein in a specific process. Here, we describe a detailed protocol for determining protein accumulation kinetics at the division site in the budding yeast *Saccharomyces cerevisiae* and fission yeast *Schizosaccharomyces pombe*. This protocol can be adapted for the analysis of any protein involved in any process as long as the protein is localized to a discrete region of the cell.

For complete details on the use and execution of this protocol, please refer to Okada et al. (2021) and Okada et al. (2019).

## Highlights

• Critical factors for live-cell imaging in yeast systems
• Detailed protocol for analyzing protein localization kinetics
• Kinetic analysis of myosin-II accumulation at the division site
• Limitations of kinetic analysis in uncovering biological mechanisms

## Graphical abstract

**Figure:**
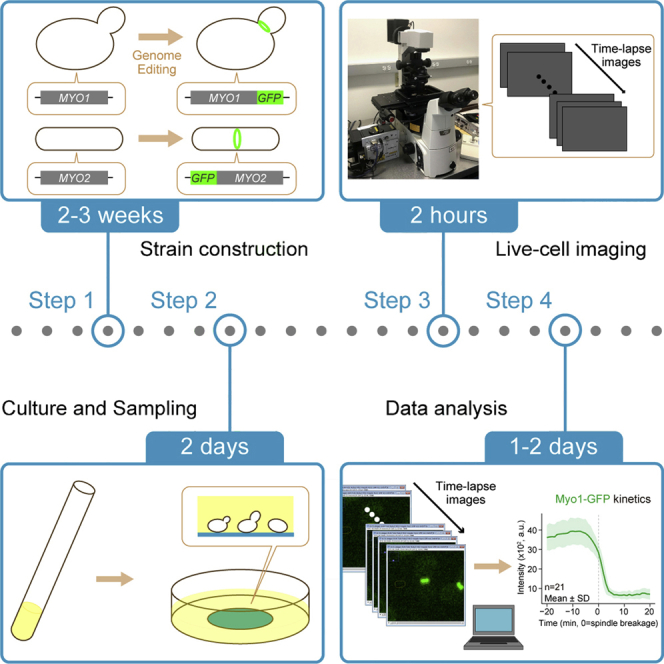

## Before you begin

The protocol below describes the specific steps used for the kinetic analysis of more than twenty different cytokinetic proteins at the division site in the budding yeast *S. cerevisiae* (Okada et al., 2021). The same protocol was also used to define the roles of myosin-II isoforms in cytokinesis under normal and stress conditions in the fission yeast *S. pombe* (Okada et al., 2019).

### Strain construction

**Timing: 2–3 weeks**

1. Introduce a cell cycle marker.
  a. The kinetic profile of a protein during the cell cycle represents data from many cells, so a cell cycle marker for aligning the individual curves of protein accumulation is essential. A tight alignment indicates data reliability. The cell cycle marker must: (1) represent a definitive cell cycle event, (2) provide an unambiguous time point for alignment, and (3) have no adverse effect on the biological process of interest. Choose an appropriate marker for your analysis.

**Figure 1:**
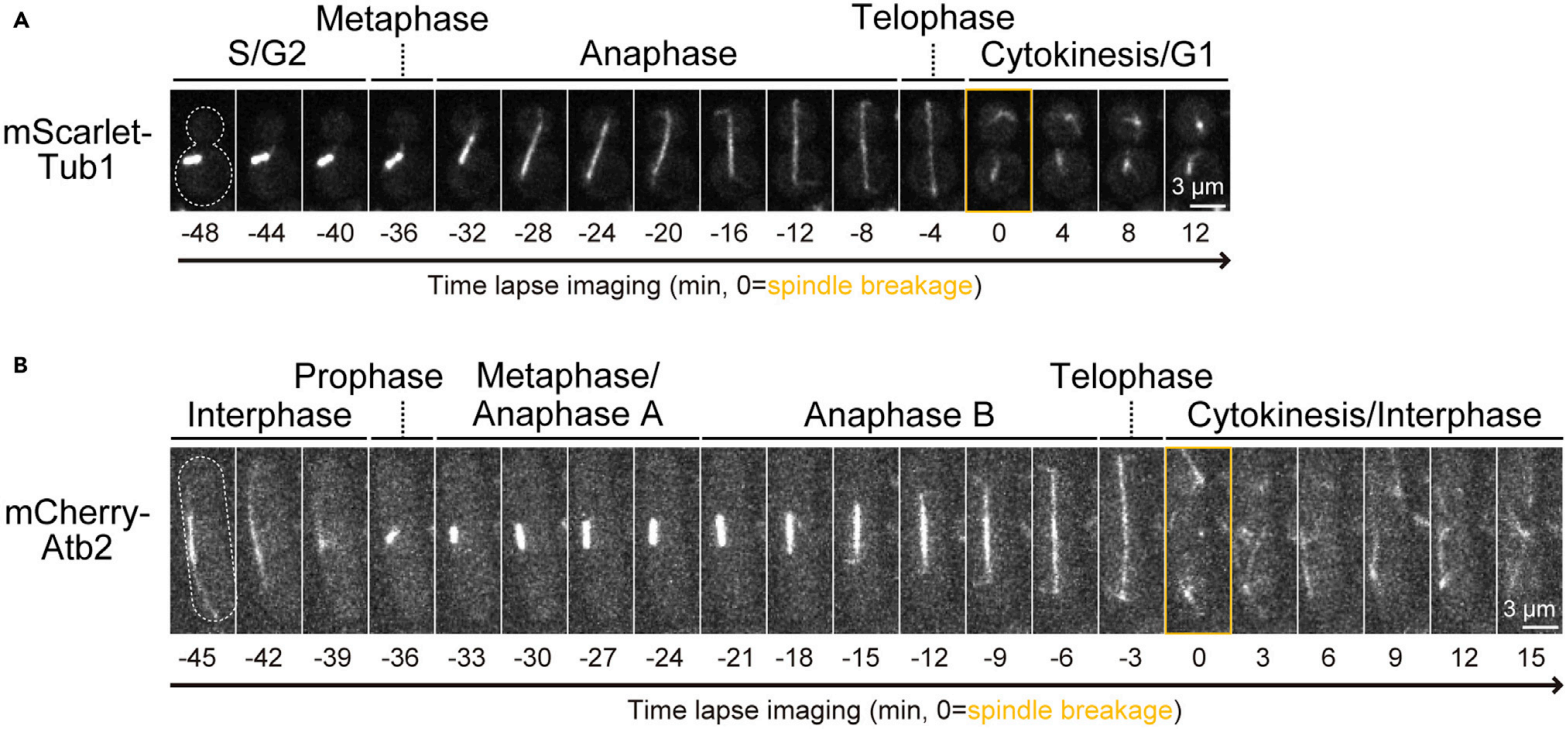
The spindle morphogenesis during the cell cycle in budding yeast and fission yeast The localization of (A) mScarlet-Tub1 in budding yeast or (B) mCherry-Atb2 in fission yeast. Montages of a representative cell from the yeast strain YEF10879 or YCW0130 are created from selected frames of time-lapse series taken with a 2-min or 1.5-min interval, respectively. The gray dotted line represents the cell outline. The images defined by the orange boxes represent spindle breakage.

***Note:*** We routinely use tubulin tagged with a fluorescent protein (FP) (e.g., RFP-Tub1 in budding yeast and mCherry-Atb2 in fission yeast) as our cell cycle marker and use the time of spindle breakage to indicate the onset of cytokinesis (Figure 1) (Okada *et al.*, 2021; Okada *et al.*, 2019).

2. Select an FP for tagging the protein.
  a. Although many FPs are available (Bialecka-Fornal et al., 2016), we prefer the classic EGFP due to its excellent brightness and photostability (Botman et al., 2019). Alternatively, GFP^E^^nvy^ can be used, as it is also bright, fast-folding, and photostable (Slubowski et al., 2015). In our hands, GFP^E^^nvy^ performed better than EGFP under certain conditions, such as high temperature (37°C). We use yeast codon-optimized FPs (e.g., yEGFP and yoEGFP) to avoid an expression bottleneck caused by rare-codon usage (Lee et al., 2013).
  b. For dual-color imaging with EGFP, we recommend ymScarlet-I, a bright and fast-folding RFP (Bindels et al., 2017).
3. Choose the location and method for tagging the endogenous gene.
  a. The N- and C-termini of proteins are usually exposed at the surface of their structures and are more distant from their structural cores (Jacob and Unger, 2007). This is why FP tags are usually placed at the N- or C-termini of the proteins. Due to its simplicity, C-terminal tagging is routinely used in budding yeast (Huh et al., 2003; Longtine et al., 1998). Another advantage is that the tagged gene is still under the control of its endogenous promoter, so protein expression is unlikely to be affected.
  b. For C-terminal tagging, the simple and efficient PCR-based method has been widely used (Longtine et al., 1998). Various methods have also been developed to allow for N-terminal tagging with native promoter control or tag insertion within an open reading frame (ORF), but these can be more time-consuming (Hawkins et al., 2003; Yofe et al., 2016). CRISPR/Cas9-based methods have become increasingly popular because they allow for tagging multiple genes simultaneously and offer more choices for tagging locations (Buchmuller et al., 2019; Torres-Garcia et al., 2020).

**CRITICAL:** For convenience and simplicity, low-copy or centromere plasmids have often been used for protein localization analysis. However, this approach is not recommended for protein kinetic analysis, as the plasmid copy number varies among individual cells, which causes a variation in the level of protein expression that might lead to changes in kinetic behavior.

4. Check for the functionality of the tagged protein.
  a. The functionality of a protein tagged with an FP can be examined by various methods, including analyzing the growth rate, morphology, or chemical sensitivity of cells carrying the tagged protein. Choose the appropriate method for your analysis. Troubleshooting 1

**CRITICAL:** It is essential to make sure that tagging does not compromise protein function. In the study of cytokinetic protein kinetics (Okada *et al.*, 2021), we co-expressed Mlc2-mApple along with the GFP-tagged cytokinetic protein in every strain, which allowed us to monitor the actomyosin ring and to ensure that the GFP-tagged proteins did not perturb actomyosin ring constriction.

### Yeast media preparation

**Timing: 2–3 h**

5. YPD agar medium (1 L, rich medium for budding yeast)
  a. Prepare two mixtures in separate flasks.
    Mixture 1

**Table:** 

Reagent	Amount
Yeast extract	10 g
Bacto peptone	20 g
Agar	20 g

Add distilled water up to 900 mL.

Put a magnetic stir bar in the flask.
    Mixture 2

**Table:** 

Reagent	Amount
Glucose	20 g

Add distilled water up to 100 mL.
  b. Autoclave both mixtures (at 121°C for 20 min).
  c. Add mixture 2 to mixture 1 with stirring by a magnetic stir bar.
  d. Leave medium at 22°C–24°C to cool off (until 50°C–55°C) and pour into Petri dishes (∼30 plates).
    ***Note:*** At the ideal temperature, the medium is not solidified but not too hot to hold the flask by hand.
    ***Note:*** During pouring, if air bubbles form on the surface of the unsolidified medium, instant searing by a burner should eliminate them.
  e. Leave plates to dry for 1–2 days at 22°C–24°C and store them at 4°C.

***Note:*** During storage, place plates in a plastic bag to prevent them from drying out.

***Note:*** The medium can be stored at 4°C for at least 6 months without a noticeable change in quality.

6. SC liquid medium (1 L, minimal medium for budding yeast)
  a. Prepare two mixtures in separate glass bottles.
    Mixture 1

**Table:** 

Reagent	Amount
Yeast nitrogen base without amino acids and with ammonium sulfate	6.7 g
Amino acid mix (Table 1)	2 g

Add distilled water up to 900 mL.

    ***Note:*** Mix all the reagents by grinding with a mortar and pestle.
    ***Note:*** The powder mix can be stored in a dark place at 22°C–24°C for at least one year without a noticeable change in quality.
    ***Optional:*** This step can be skipped if you purchase a premixed powder (#1300-030, Sunrise Science, TN, USA).
    ***Optional:*** If your protein of interest is tagged with GFP and exposed to extracellular space, the GFP signal will be quenched due to the acidity of SC medium (pH ∼4.5) (Colman-Lerner et al., 2001; Thomas et al., 2018). To resolve this issue, adjust the mixture to pH 7 with sodium hydroxide solution.
    **Table 1:** Recipe for an amino acid mix (93.4 g)

Reagent	Amount
4-aminobenzoic acid	0.4 g
Adenine	1 g
Alanine	4 g
Arginine	4 g
Asparagine	4 g
Aspartic acid	4 g
Cysteine	4 g
Glutamic acid	4 g
Glutamine	4 g
Glycine	4 g
Histidine	4 g
Isoleucine	4 g
Leucine	8 g
Lysine	4 g
Methionine	4 g
myo-Inositol	4 g
Phenylalanine	4 g
Proline	4 g
Serine	4 g
Threonine	4 g
Tryptophan	4 g
Tyrosine	4 g
Uracil	4 g
Valine	4 g
    Mixture 2

**Table:** 

Reagent	Amount
Glucose	20 g

Add distilled water up to 100 mL.
  b. Autoclave both mixtures (at 121°C for 20 min).
  c. Leave medium at 22°C–24°C to cool off. Store in a dark place.
    ***Note:*** The medium can be stored at 22°C–24°C for at least three months without a noticeable change in quality.
  d. Just before use, mix two solutions (mixture 1:mixture 2 = 9:1).

***Optional:*** You can mix the entirety of the two solutions immediately after autoclaving, but this increases the risk of contamination.

7. YE5S agar or liquid medium (1 L, rich medium for fission yeast)
  a. Prepare the mixture in a flask (for agar medium) or bottle (for liquid medium).

**Table:** 

Reagent	Amount
Yeast extract	5 g
Glucose	30 g
Adenine	0.2 g
Uracil	0.2 g
Histidine	0.2 g
Leucine	0.2 g
Lysine	0.2 g
Agar (for agar medium only)	20 g

Add distilled water up to 1 L.
  b. Autoclave the mixture (at 121°C for 20 min).
  c. (If agar medium) Leave medium at 22°C–24°C to cool off (until 50°C–55°C) and pour into Petri dishes (∼30 plates). Dry plates for 1–2 days at 22°C–24°C and store them at 4°C.
  d. (If liquid medium) Leave medium at 22°C–24°C to cool off and store at 22°C–24°C.

***Note:*** The medium can be stored for at least 3 months without a noticeable change in quality.

### Concanavalin A (Con A)/lectin coating of imaging dish

**Timing: 30–60 min**

8. Prepare the stock solution.
  a. Dissolve Con A (2 mg/mL) in distilled water with 5 mM MnCl_2_ and 5 mM CaCl_2_. Store aliquots (1 mL) at −20°C.

***Note:*** Frozen aliquots can be stored for several years without a noticeable loss of efficiency.

9. Con A coating
  a. Thaw Con A solution and place 100–150 μL on the glass bottom of the dish.
  b. Incubate for 15 min at 22°C–24°C and remove the Con A solution.
    ***Note:*** Collected Con A solution can be reused at least 5 times.
  c. Wash by vigorous pipetting with 200 μL of distilled water. Repeat this step 5 times.
  d. Remove water and leave the dish until the remaining water evaporates completely. Put the lid on to avoid dust falling into the dish.
  e. Store the coated dish at 4°C.

**CRITICAL:** For the fission yeast protocol, replace Con A with lectin (L1395, Sigma, MA, USA) because Con A does not provide efficient adhesion to fission yeast.

***Optional:*** This step can be skipped if you purchase a precoated dish (BioMedTech Laboratories, FL, USA).

***Note:*** Coated dishes can be stored for at least 6 months without noticeable loss of efficiency.

## Key resources table

**Table:** 

REAGENT or RESOURCE	SOURCE	IDENTIFIER
**Chemicals, peptides, and recombinant proteins**		

Adenine	Acros Organics	Cat#: 16363-1000
Agar	Fisher Scientific	Cat#: BP1423-500
Bacto peptone	BD Biosciences	Cat#: 211677
L-Alanine	Fisher Scientific	Cat#: BP369-100
L-Arginine	Fisher Scientific	Cat#: BP372-100
L-Asparagine	Fisher Scientific	Cat#: BP373-100
L-Aspartic Acid	Fisher Scientific	Cat#: BP374-100
Calcium Chloride, Dihydrate	Fisher Scientific	Cat#: C79-500
L-Cysteine	Fisher Scientific	Cat#: BP376-100
Concanavalin A	Millipore Sigma	Cat#: L7647
D-Glucose	Fisher Scientific	Cat#: D16-1
L-Glutamic Acid	Fisher Scientific	Cat#: BP378-100
L-Glutamine	Fisher Scientific	Cat#: BP379-100
Glycine	Fisher Scientific	Cat#: G46-500
L-Histidine	Fisher Scientific	Cat#: BP382-100
L-Isoleucine	Fisher Scientific	Cat#: BP384-100
L-Leucine	Fisher Scientific	Cat#: BP385-100
Lectin from Glycine max (soybean)	Millipore Sigma	Cat#: L1395
L-Lysine	Fisher Scientific	Cat#: BP386-100
L-Methionine	Fisher Scientific	Cat#: BP388-100
Manganese Chloride Tetrahydrate	Fisher Scientific	Cat#: M87-100
myo-Inositol	Millipore Sigma	Cat#: I5125
L-Phenylalanine	Fisher Scientific	Cat#: BP391-100
L-Proline	Fisher Scientific	Cat#: BP392-100
L-Serine	Fisher Scientific	Cat#: BP393-100
L-Threonine	Fisher Scientific	Cat#: BP394-100
L-Tryptophan	Fisher Scientific	Cat#: BP395-100
L-Tyrosine	Fisher Scientific	Cat#: BP396-100
Uracil	Millipore Sigma	Cat#: U0750
L-Valine	Fisher Scientific	Cat#: BP397-100
Yeast extract	Fisher Scientific	Cat#: BP1422-500
Yeast nitrogen base without amino acids	Research Products International	Cat#: Y20040–250.0
4-Aminobenzoic acid	Acros Organics	Cat#: 14621-2500

**Deposited data**		

Example imaging dataset (Myo1-GFP)	Mendeley Data	https://dx.doi.org/10.17632/sv9d9hmvrm.1

**Experimental models: Organisms/strains**		

*S. cerevisiae*: Strain YEF9609: *MATa trp1-Δ63 leu2-Δ1 ura3-52 his3-Δ200 lys2-801 TUB1::HPH-pHIS3-mRuby2-TUB1 MLC2-mApple-URA3MX MYO1-GFP-His3MX6*	(Okada et al., 2021)	N/A
*S. cerevisiae*: Strain YEF10879: *MATα his3Δ1 leu2Δ0 lys2Δ0 ura3Δ0 TUB1::HPH-pHIS3-ymScarlet-I-TUB1 GFP*^*Envy*^*-EGT2*	This study	N/A
*S. pombe*: Strain YCW0130: *h*^*-*^*kanMX6-Pmyo2-mEGFP-myo2 ade6-M210 leu1-32 ura4-D18 mCherry-atb2:hphMX6*	(Okada et al., 2019)	N/A

**Software and algorithms**		

Fiji	(Schindelin et al., 2012)	N/A
MetaMorph, version 7.8.10.0	Molecular Devices	N/A
Microsoft Excel	Microsoft	N/A
NIH ImageJ (1.51j)	(Schindelin et al., 2012)	N/A
R (ver 3.0.1)	The R Project for Statistical Computing	https://www.r-project.org/

**Other**		

4-Chamber 35-mm glass bottom dish	Cellvis	Cat#: D35C4-20-1.5-N
Sonicator	Qsonica	Q55
Water bath shaker	New Brunswick Scientific	Model G76
Spectrophotometer	Thermo Scientific	NanoDrop 2000c
Microscope	Nikon	Eclipse Ti-U
Objective lens	Nikon	CFI Apo TIRF 100**×**/1.49NA
Light source	Spectral Applied Research	ILE-400
EMCCD camera	Photometrics	Evolve 512 Delta
Confocal system	Yokogawa	CSU-X1

## Materials and equipment

To perform analysis of protein accumulation kinetics in yeast cells, a spinning-disk confocal microscope is the first choice due to its fast acquisition ability with minimal photobleaching and phototoxicity. Details of the suitable device configuration are described previously (Okada et al., 2017). See the key resource table under the heading "Other" for the devices used for the imaging examples shown in this protocol.

## Step-by-step method details

### Preparation of cell culture

**Timing: 2–3 days**

Yeasts were typically stored in glycerol stock at −80°C, and this step explains how to revitalize frozen cells in preparation for live-cell imaging.

1. Streak out the strain(s) of interest on YPD plates. Incubate them for 1–2 days at 25°C or 30°C.

***Note:*** Use YE5S plates for fission yeast.

***Note:*** Cells on rich media can survive for 3–4 weeks at 4°C. To prevent the plates from drying out, they should be sealed with parafilm. Minimal media are not recommended for storage purposes because cells on such media are prone to lose viability more quickly.

2. A day before the planned imaging, inoculate cells from a single colony on the plate (step 1) into 4 mL of SC liquid medium in a test tube (16 **×** 150 mm, round bottom) and shake them at 25°C for 6–8 h in a water bath shaker (at 210 rpm).

***Note:*** For fission yeast, we use 8 mL of YE5S liquid medium in a test tube (25 **×** 120 mm, flat bottom).

**CRITICAL:** YPD liquid medium should be avoided for imaging purposes due to its high background of autofluorescence.

***Optional:*** As an alternative, make a thin cell patch on a fresh plate from a single colony and then incubate at 25°C. This also works sufficiently well.

3. Transfer preculture (inoculation volume needs to be calculated, see note) to 4 mL of SC medium in a test tube (16 **×** 150 mm, round bottom) and shake them at 25°C overnight for 12–16 h in a water bath shaker (at 210 rpm). Cells are cultured until they reach the mid-log phase (OD_600_ = 0.3–0.7, 0.4–1 × 10^7^ cells/mL).

***Note:*** For fission yeast, we use 8 mL of YE5S liquid medium in a test tube (25 **×** 120 mm, flat bottom).

***Note:*** Use the spreadsheet to calculate the inoculation volume (See Table S1)

***Optional:*** To increase the likelihood of capturing cells at the right phase of growth for imaging, prepare multiple cultures by serial dilution to gain a broader range of cell density at the time of collection.

### Live-cell imaging

**Timing: 3–4 h**

This step explains how to prepare the sample and acquire time-lapse images.

4. Sample preparation
  a. Transfer 1 mL of mid-log culture to a 1.5 mL microcentrifuge tube.
    ***Note:*** Use a low retention tube for harvesting cells because cells in a minimal medium tend to adhere to the tube's inner wall and are not pelleted efficiently in a regular tube.
  b. Declump cells by sonication for 5 s at 15% power (Q55, Qsonica).
    ***Note:*** The sonication step can be skipped if cells do not form aggregates.
  c. Concentrate cells by centrifugation (8,000 **×** *g*) and remove the supernatant.
  d. Resuspend the pellet to 150 μL fresh SC medium.
    ***Note:*** Use YE5S medium for fission yeast.
  e. Place the entire cell suspension to the Con A/Lectin-coated glass-bottom dish.
  f. Mount the dish on the microscope stage and incubate 5–15 min to sediment the cells. During cell sedimentation, check the cell density on the glass bottom occasionally by microscope. Troubleshooting 2
  g. When the cell density reaches an appropriate level (see details below), gently remove the supernatant and wash cells with 200 μL fresh SC medium. Repeat the wash step 2–3 times to get rid of floating and unattached cells. Gently remove the supernatant and add 1–1.5 mL of SC medium.

***Note:*** Use YE5S medium for fission yeast.

***Note:*** While changing medium, leave a small amount of supernatant to prevent cells from drying out.

***Note:*** Indicated medium volume (1–1.5 mL) is for a single chamber in the 4-chamber 35 mm dish (D35C4-20-1.5-N, Cellvis). If you use a regular non-chambered 35 mm dish, add 4–5 mL to provide cells with sufficient nutrition.

**Figure 2:**
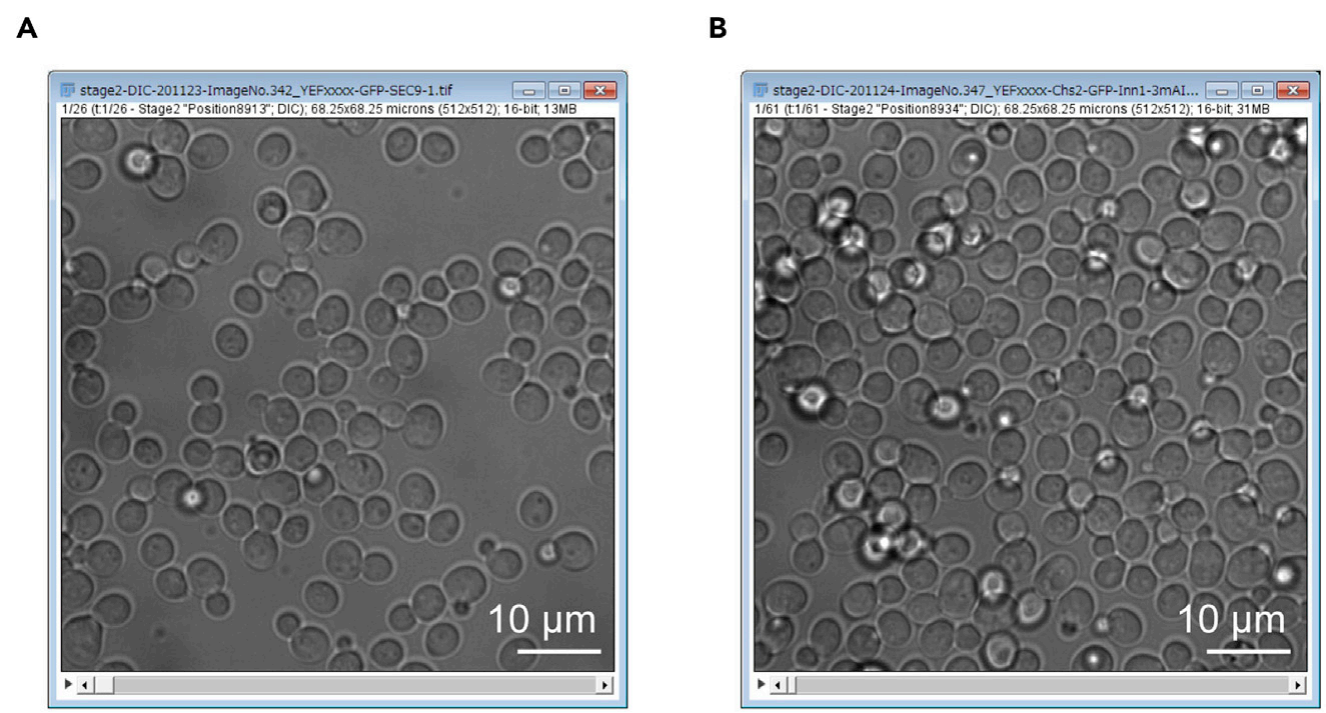
Examples of cell densities in a microscopic field for imaging in budding yeast (A) A field of cells at an appropriate density for analysis (50–70% coverage). (B) A field of cells at an excessive density for analysis (90–100% coverage).

**CRITICAL:** We prefer the cell density at 50–70% field coverage by cells (Figure 2A). Overcrowded fields (90–100% field coverage, Figure 2B) should be avoided because the cell-free area will be used as a background reference in the following data analysis.

***Optional:*** We recommend this preparation method because it is simple and does not require special equipment or reagents. This method can keep cells healthy for prolonged imaging times (>2 h) and allows for drug perfusion. There are many alternative ways to prepare specimens for live-cell imaging, e.g., using an agarose pad (Okada *et al.*, 2017). If you need special conditions, such as accurate temperature regulation, you should select the appropriate method for your experiment (Babic et al., 2018).

5. Time-lapse imaging
  a. These are typical settings for our routine imaging (Table 2). The conditions should be adjusted to be appropriate for the protein of interest and your imaging system. Troubleshooting 3, 4, and 5

**Table 2:** Settings for imaging

GFP excitation/emission	RFP excitation/emission	Laser power	Exposure time	EM gain	Z-stack	Imaging duration	Time interval
488/505 nm	561/595 nm	3–5%	30–100 ms	200–300	11 sections with 1.0 μm spacing	60–90 min	1 or 1.5 min

## Expected outcomes

**Figure 3:**
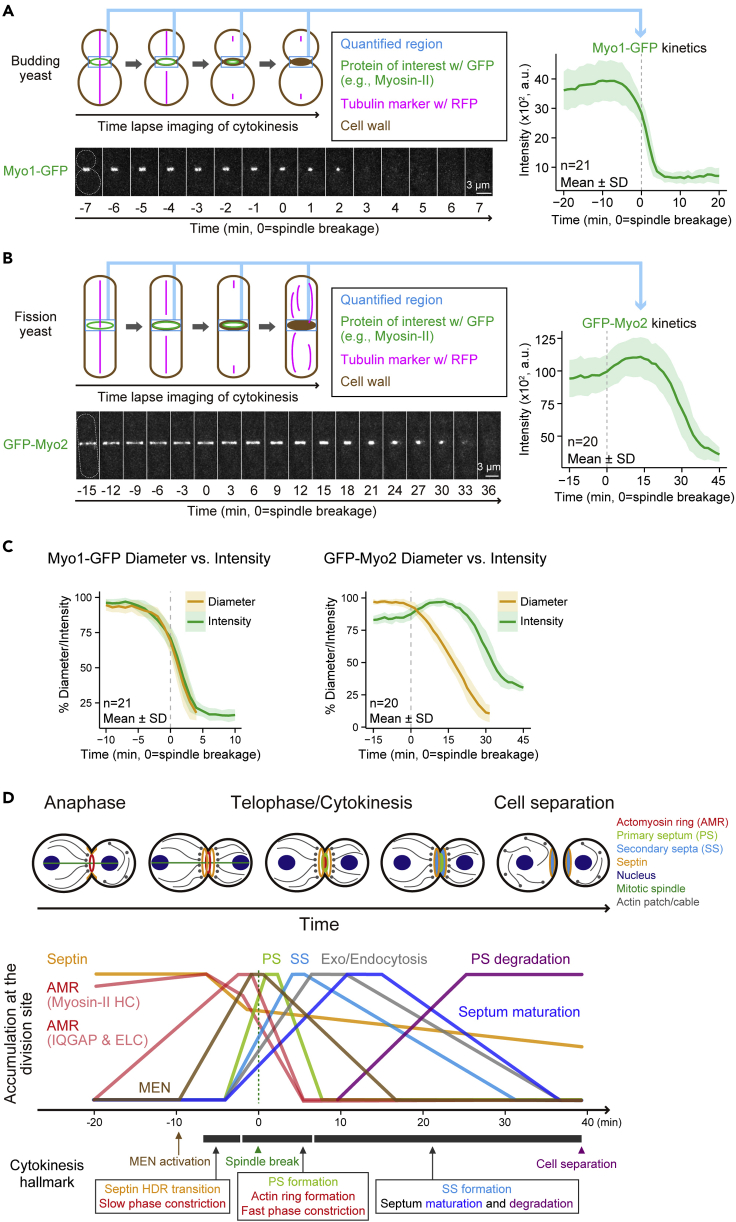
Quantification of myosin-II kinetics at the division site (A) Kinetic analysis of the sole myosin-II (Myo1-GFP) in budding yeast. Fluorescent intensity of Myo1-GFP at the division site (blue box) of the budding yeast strain YEF9609 was measured from a series of time-lapse images. Montages of a representative cell were created from selected frames of time-lapse series taken with a 1-min interval. The gray dotted line represents the cell outline. All data from individual cells were aligned in relation to the timing of mitotic spindle breakage. Bold lines and associated shaded bands represent mean and s.d. values, respectively. Modified and reproduced from Okada et al., 2021. (B) Kinetic analysis of the essential myosin-II (GFP-Myo2) in fission yeast. Time-lapse imaging and data analysis of the fission yeast strain YCW0130 were conducted as described in (A) with slight modifications to fit for fission yeast (see text and note for details). Similar results were published in Okada et al., 2019. (C) Diameter and intensity of Myo1-GFP and GFP-Myo2 during constriction. Plots were created from the data in (A) and (B). For Myo1-GFP, reproduced from Okada et al., 2021; for GFP-Myo2, similar data were published in Okada et al., 2019. (D) The kinetic landscape of cytokinesis. As the cell cycle progresses from the onset of mitotic exit to cell separation, distinct functional modules accumulate at the division site in a strict temporal order to carry out their specific roles in cytokinesis. Reproduced from Okada et al., 2021.

By following the steps described above and the quantification and statistical analysis described below, you can acquire local protein accumulation kinetics. Figure 3 shows an analysis scheme and the kinetics of myosin-II at the division site during cytokinesis in both budding yeast (Myo1-GFP, Figure 3A) and fission yeast (GFP-Myo2, Figure 3B). Accumulation kinetics are an information-rich measurement. Different aspects of a kinetic curve, including the specific rate of rise or fall and the peak accumulation time, could reveal different molecular behaviors of a protein, including its functional time, a localization switch, or a potential structural transition during the cell cycle. Kinetic analysis of cytokinetic proteins at the system level has led to some general rules that can be used to interpret or predict protein behavior (Okada et al., 2021). For example, proteins sharing similar kinetics might reflect similar molecular functions and/or functioning in the same complex. In addition, mechanistic insights into distinct behaviors of different members of a protein family can be obtained by kinetic analysis. For example, the disappearance pattern of myosin-II in budding yeast (Myo1-GFP), but not in fission yeast (GFP-Myo2), accurately reflects the kinetics of actomyosin ring constriction during cytokinesis (Figure 3C). Moreover, the plot created by superimposing the kinetics of many different proteins is exceptionally informative. The analysis of more than twenty proteins has depicted a kinetic landscape of cytokinesis, revealing a strict temporal regulation of budding yeast cytokinesis (Figure 3D).

## Quantification and statistical analysis

To establish the kinetics of protein accumulation from a sequence of cell images, we need to extract the temporal changes in protein intensity at a specific subcellular location. This step describes how to analyze protein kinetics using our published protocol (Okada et al., 2017) with a slight modification. The example datasets are deposited at Mendeley Data: https://dx.doi.org/10.17632/sv9d9hmvrm.1.

1. Import and process an image sequence
  a. Open the image sequence (*File>Open*, select nd file) in Fiji (Schindelin et al., 2012) and create a z-stack of images by combining a series of images taken at different focal planes using sum projections (*Image>Stacks>Z Project,* choose *sum slices* for projection type).
  b. Apply the StackReg plugin (Thevenaz et al., 1998, http://bigwww.epfl.ch/thevenaz/stackreg/) to align the stack of images (*Plugins>Registration>StackReg*, choose *Rigid Body* for transformation).

***Note:*** StackReg takes quite a long time if your stack is large and contains many slices. Run the plugin on a computer with a powerful processor. For example, processing of one z-stack (512**×**512 pixels, 55 slices) takes ∼15 seconds by our workstation (CPU: Intel Xeon W-2223 3.60GHz), while ∼30 seconds by our laptop (CPU: Intel Core i5-2410M 2.30GHz).

**Figure 4:**
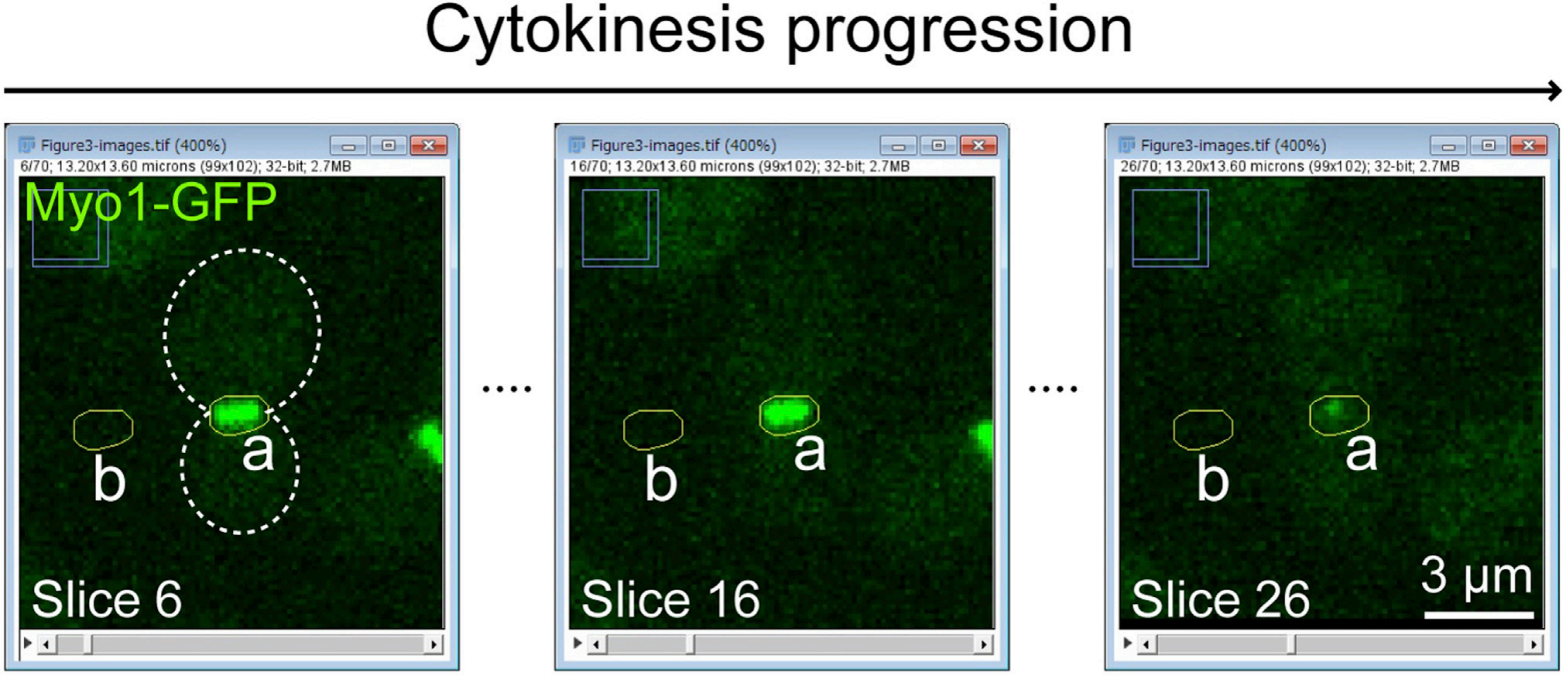
Drawing an appropriate ROI for the quantification of Myo1-GFP kinetics during cytokinesis Please note that the ROIs (yellow polygons) for the protein of interest (Myo1-GFP, "a") and for the background ("b") must cover the intended locations throughout the process of cytokinesis. The gray dotted line represents the cell outline.

**Figure 5:**
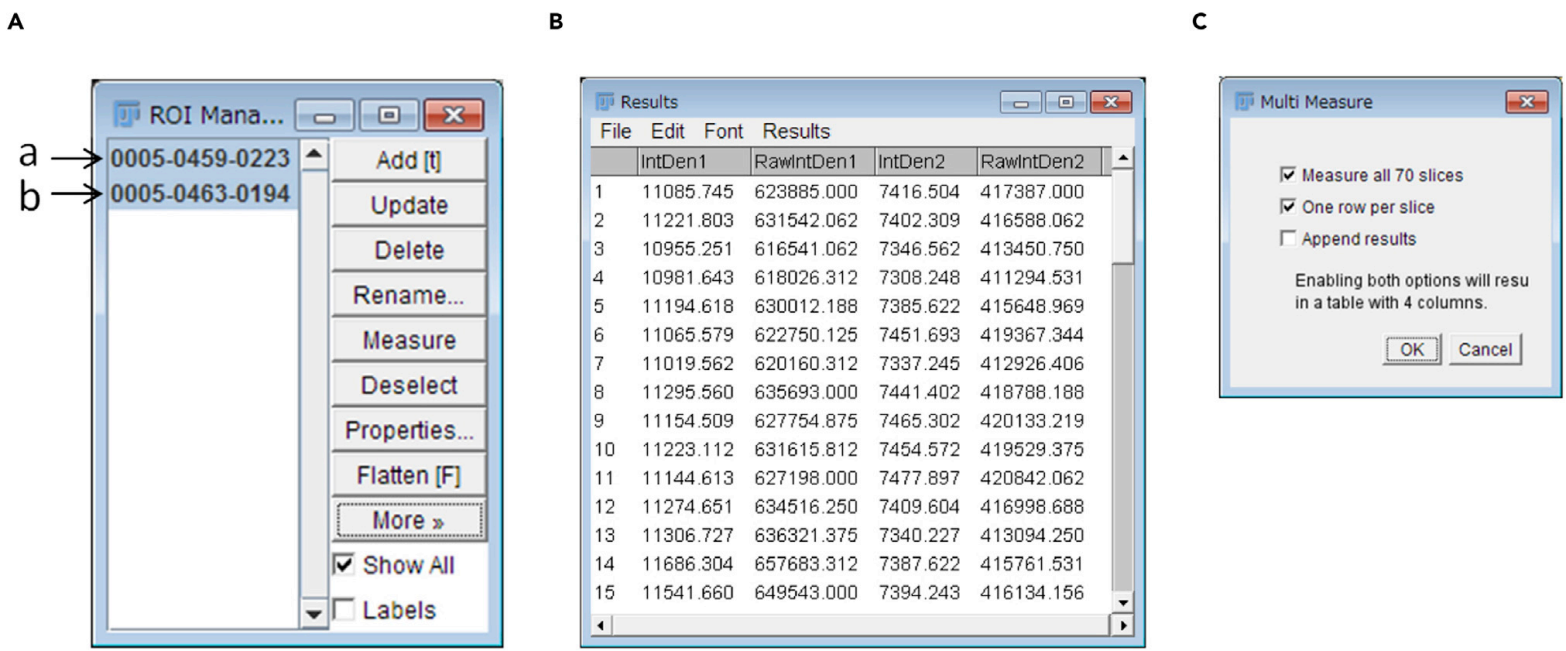
Configuration and quantification (A) Make sure that the two ROIs ("a" and "b") intended for quantification are selected (in blue highlight). (B) Example of measurement results. (C) Make sure that the two boxes are checked before running a measurement.
    ***Note:*** Ensure that the polygon contains the ROI throughout the biological process of interest by scrolling through the image slices. Troubleshooting 4
    ***Note:*** In this example, we monitored myosin-II (marked by Myo1-GFP) until the end of cytokinesis.
  b. Click and drag the polygon to the background area ("b" in Figure 4) and add it to the ROI manager.

***Note:*** Ensure that the polygon stays in the background area throughout the biological process of interest by scrolling through the image slices.

2. Selection of the ROI (region of interest)
  a. Draw a polygon (click *Polygon selections* in the toolbar) around the ROI ("a" in Figure 4) and add it to ROI manager (*Edit>Selection>Add to Manager* or press the T key on a keyboard) (Figure 5A).

***Optional:*** Using a cell-free area as the background may artificially increase the signal for the protein of interest at a subcellular location. To avoid this problem, some researchers might prefer to use the signal in an intracellular area as the background. However, yeast cytosol is not uniform in composition due to the movement of organelles during the cell cycle. In addition, the cytosolic concentrations of some proteins may change as the result of changes in their expression and/or localization during the cell cycle. These factors would increase the signal fluctuations in the background, thus, rendering this alternative less favorable.

**Figure 6:**
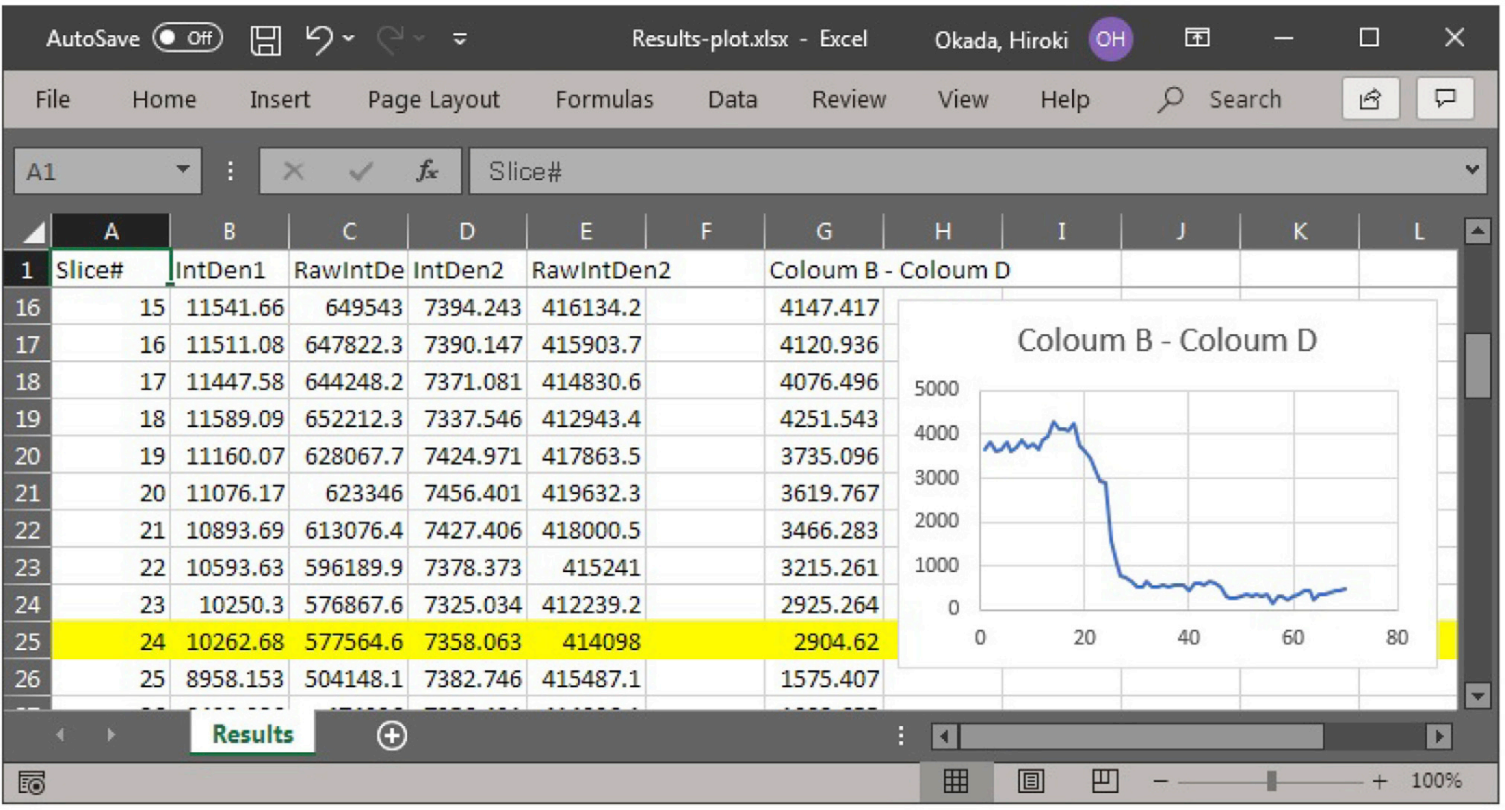
Visualization of the result from a single ROI by Microsoft Excel Fluorescent intensity of the ROI is visualized by line plot after subtraction of background intensity.

**Figure 7:**
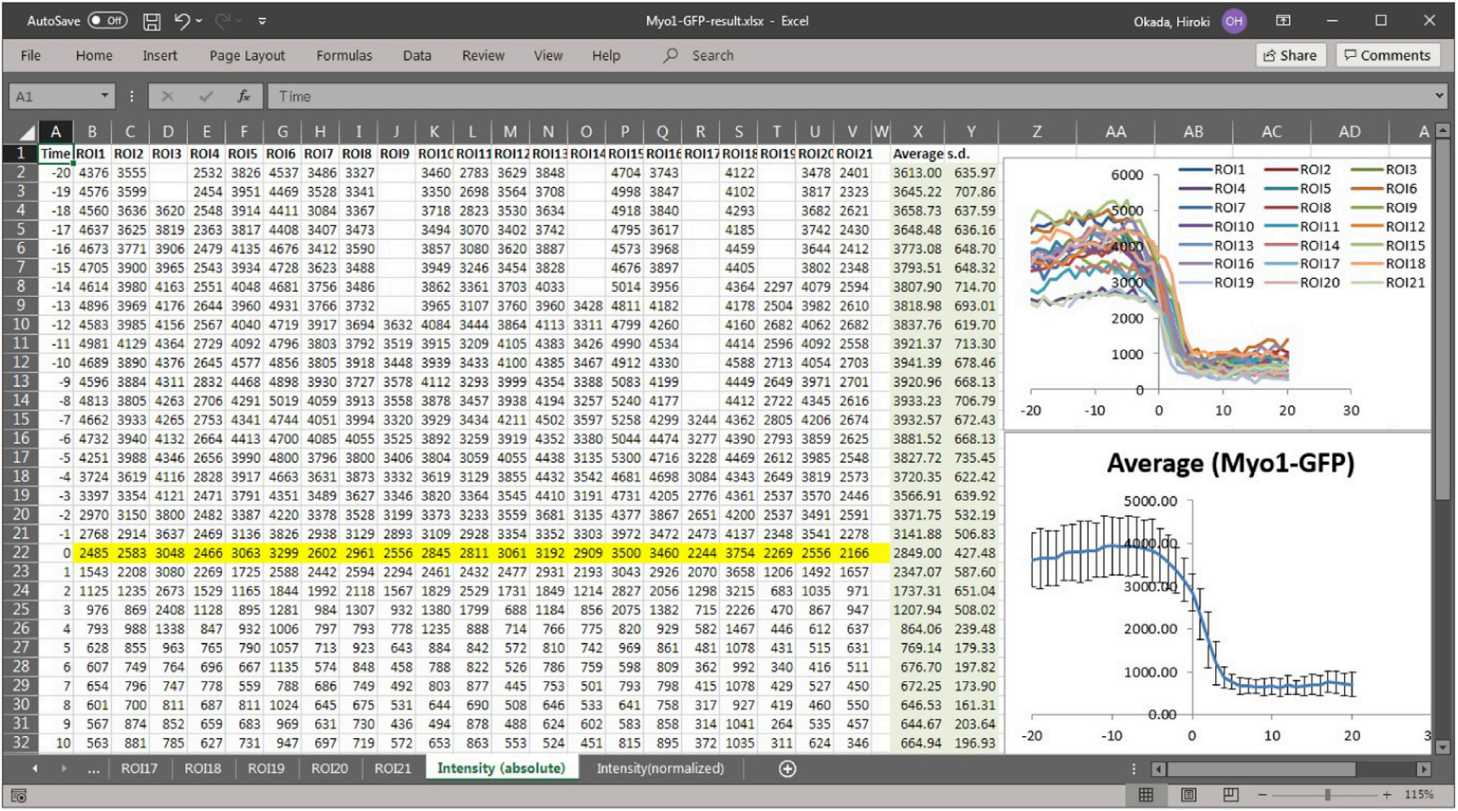
Summarizing the result from multiple ROIs It is informative to draw both a summarized chart (bottom-right, mean and s.d.) and all individual plots (top-right) to inspect data quality and reliability.

3. Perform measurement and data visualization.
  a. Ensure that the integrated density box is checked in the measurements (*Analyze>Set Measurements*).
  b. In the ROI manager, select both ROIs ("a" and "b" from step 2; click + Shift key, Figures 4 and 5A) and perform Multi Measure (*More>Multi Measure*). Results will appear in a pop-up window (Figure 5B).
    ***Note:*** Ensure that the two boxes (*Measure all x slices* and *One row per slice*) are checked (Figure 5C).
  c. Save and open the results in Excel or a statistical program, such as GraphPad or R. To measure the protein intensity, subtract the background integrated density (*IntDen2* measured from the ROI "b", Figure 4) from the integrated density of the ROI of the biological process (*IntDen1* measured from the ROI "a", Figure 4). To review the data, plot the changes of fluorescent intensity over time (Figure 6).

4. Generate a summary of the results by aligning the individual plots.
  a. For each dataset from a specific ROI, mark the image number (yellow highlight in Figure 6) in which a cell cycle event can be used as a reference point for plot analysis.
    ***Note:*** In this example, we used spindle (marked by RFP-Tub1, Figure 1) breakage monitored in a different channel as the reference point (time "0" for the x-axis).
  b. Align all the data from individual ROIs by the marked slice as time 0, and plot summarized data, such as mean with standard deviation (Figure 7).

## Limitations

While kinetic analysis of protein accumulation at a specific subcellular location provides a significant amount of information about the function of a protein in a real-time biological process, such an analysis alone is not adequate for defining the mechanism of action for that specific protein. Kinetic analysis must be combined with genetic and/or biochemical analysis to draw any meaningful conclusions.

## Troubleshooting

### Problem 1

Compromised function caused by FP tagging (step 4 in Before you begin)

### Potential solution

The difference in FP could result in a difference in phenotype (Jiang et al., 2017). Consistent with this, we observed that mNeonGreen tagging of Myo1 caused an FP-specific growth defect in budding yeast. Consider changing the FP if a similar problem is encountered during strain construction.

Although C-terminal tagging has been widely used, it might compromise the localization and/or function of some proteins. For example, Ras2 in budding yeast requires C-terminal modification for its targeting to the plasma membrane (Bhattacharya et al., 1995). In such cases, N-terminal tagging or tag insertion within the ORF should be attempted. For example, FP insertion at a specific site within the *cdc42*^*+*^ ORF in fission yeast does not compromise its function (Bendezu et al., 2015). Examining relevant literature and GFP-based localization databases, such as Yeast RGB (http://shmoo.weizmann.ac.il/elevy/YeastRGB/HTML/YeastRGB.html) (Dubreuil et al., 2019), is informative and helpful for deciding the best location for tagging a specific gene.

### Problem 2

Insufficient or overcrowded cell density in a microscopic field (step 4 in step-by-step method details).

### Potential solution

If cell density in the microscopic field has not reached adequate coverage during the incubation, you can add more cell suspension to the imaging dish. If the cell density is too high, you can strip cells off by pipetting.

### Problem 3

Insufficient signal intensity (step 5 in step-by-step method details).

### Potential solution

Optimize imaging conditions. To have a better signal-to-noise ratio, you can try increasing the laser power. However, this can cause adverse effects, such as enhancing photobleaching/phototoxicity. You can also change other imaging conditions, such as reducing the number of z-sections and/or setting a longer imaging interval. It also helps if you can upgrade your equipment, such as a more sensitive camera and an optimal filter cube.

Change in strain construction. Different FPs possess different signal intensities. Switching to a brighter FP (e.g., mNeonGreen) is the first thing to try. To increase the fluorescence intensity per molecule, tag your protein of interest with tandem copies (2**×** or 3**×**) of an FP. Alternatively, to increase the number of molecules per cell, change the promoter to a stronger one (e.g., *ADH1* promoter). This is done only when the pattern of protein localization is not affected by its expression level. However, it is worth noting that these strategies may also increase the probability of compromising protein function and/or introducing an artefact. Thus, it is essential to ensure that the biological process of interest is not affected by the molecular modifications.

### Problem 4

Field drift (step 5 in step-by-step method details and step 2 in quantification and statistical analysis).

### Potential solution

If it is x-y drift, image processing by the StackReg plugin can reduce the drift effect. If the StackReg does not solve the issue, it is possible to manually move the ROI to trace the location for the analysis. When changing the location of the polygon, make sure that the polygon's size and shape are not changed. If it is z drift, it is difficult to fix the problem through post-acquisition image processing. Thus, make sure that your focal plane is in the appropriate position during time-lapse imaging and, if necessary, adjust the focal position between acquisitions. Deploying an autofocus system will certainly help. We noticed that turning on the imaging system 1–2 h before imaging significantly improves the stability of the focal plane.

### Problem 5

Unhealthy cells (step 5 in step-by-step method details).

### Potential solution

Cells could slow down growth and lose viability during imaging because of the phototoxicity caused by light illumination. To reduce the toxicity, use minimal laser power and/or change an FP, which requires a longer wavelength for excitation. Differences in specimen preparation also affect cell health. We noticed that some other documented methods (e.g., agarose pad) are unsuitable for more prolonged time-lapse imaging, probably due to insufficient nutrition and/or gas exchange in the culture medium. Cell freshness is a critical factor for cell health. Thus, use freshly streaked-out cells for your experiments.

## Resource availability

### Lead contact

Further information and requests for resources and reagents should be directed to and will be fulfilled by the lead contact, Erfei Bi (ebi@pennmedicine.upenn.edu).

### Materials availability

Reagents used in the Expected Outcomes are available upon request.

## Data Availability

The example datasets generated in this study are deposited at Mendeley Data: https://dx.doi.org/10.17632/sv9d9hmvrm.1.

## Supplemental information

Table S1. Inoculation volume table, relates to step 3 of step-by-step method details
